# Supplementary figures and images for: Community-acquired pneumonia identification from electronic health records in the absence of a gold standard: A Bayesian latent class analysis
Source: PLOS Digit Health. 2025 Jul 21;4(7):e0000936. doi: 10.1371/journal.pdig.0000936 (PMC12279105; doi:10.1371/journal.pdig.0000936)

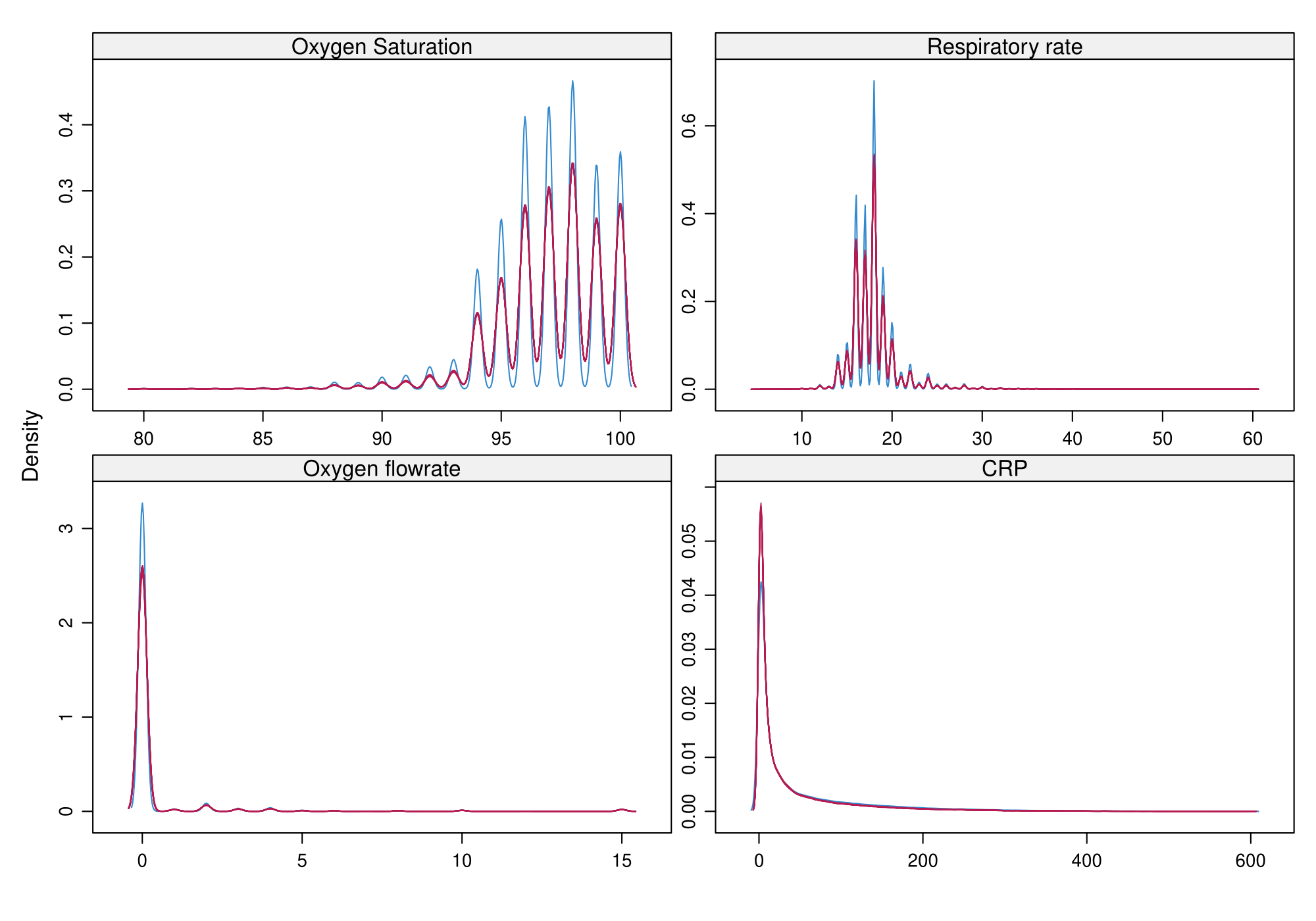

Supplement: S1 Fig — 10 imputed datasets were generated. CRP: C-reactive protein. (TIFF) [file pdig.0000936.s001.tiff]

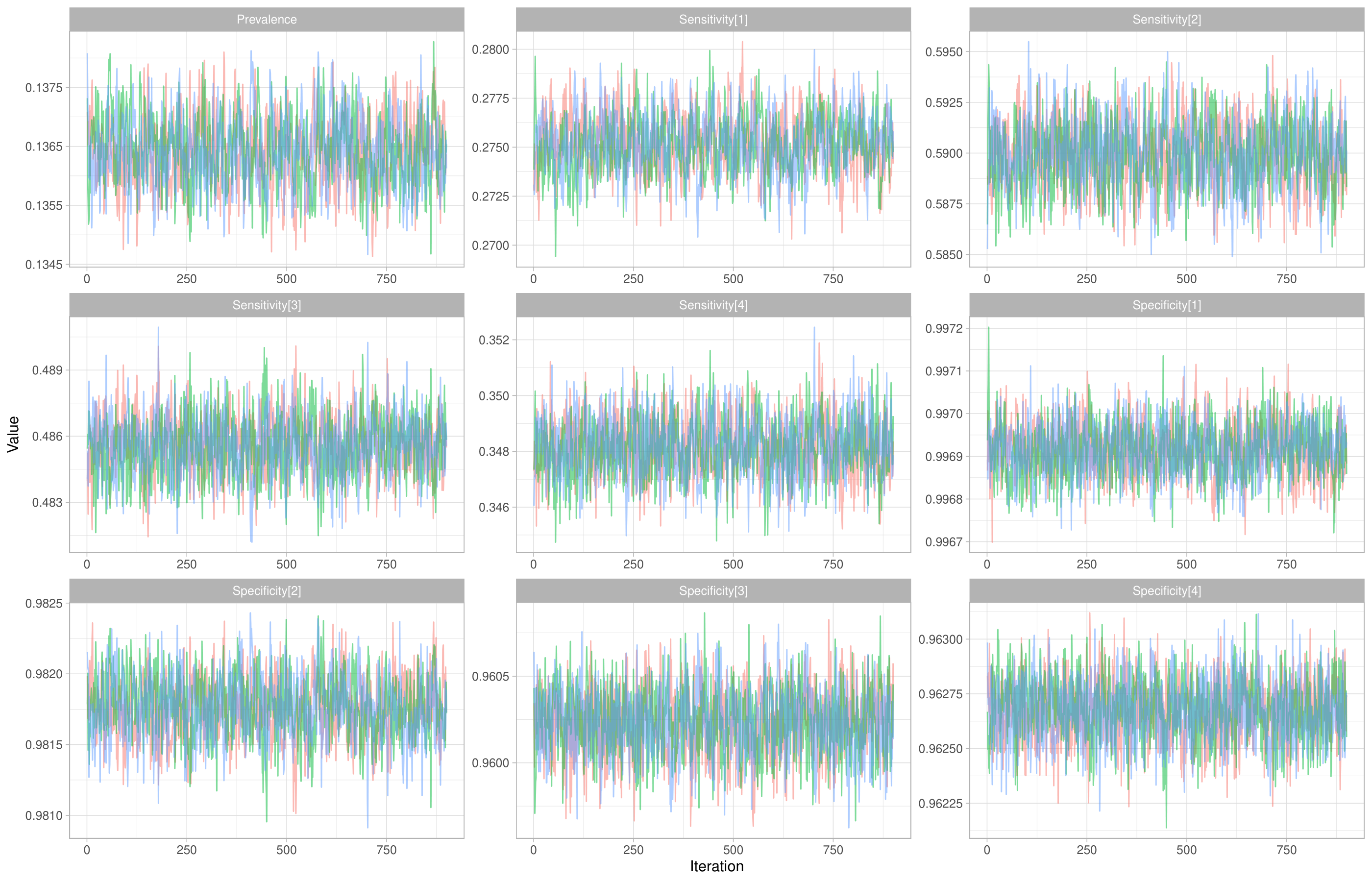

Supplement: S2 Fig — Algorithm [1] is CAP primary diagnostic codes; Algorithm [2] is CAP antibiotic indication; Algorithm [3] is chest X-ray report or CT scan report; Algorithm [4] is shortness of breath and elevated C-reactive protein levels. Trace plots indicate good convergence. (TIFF) [file pdig.0000936.s002.tiff]

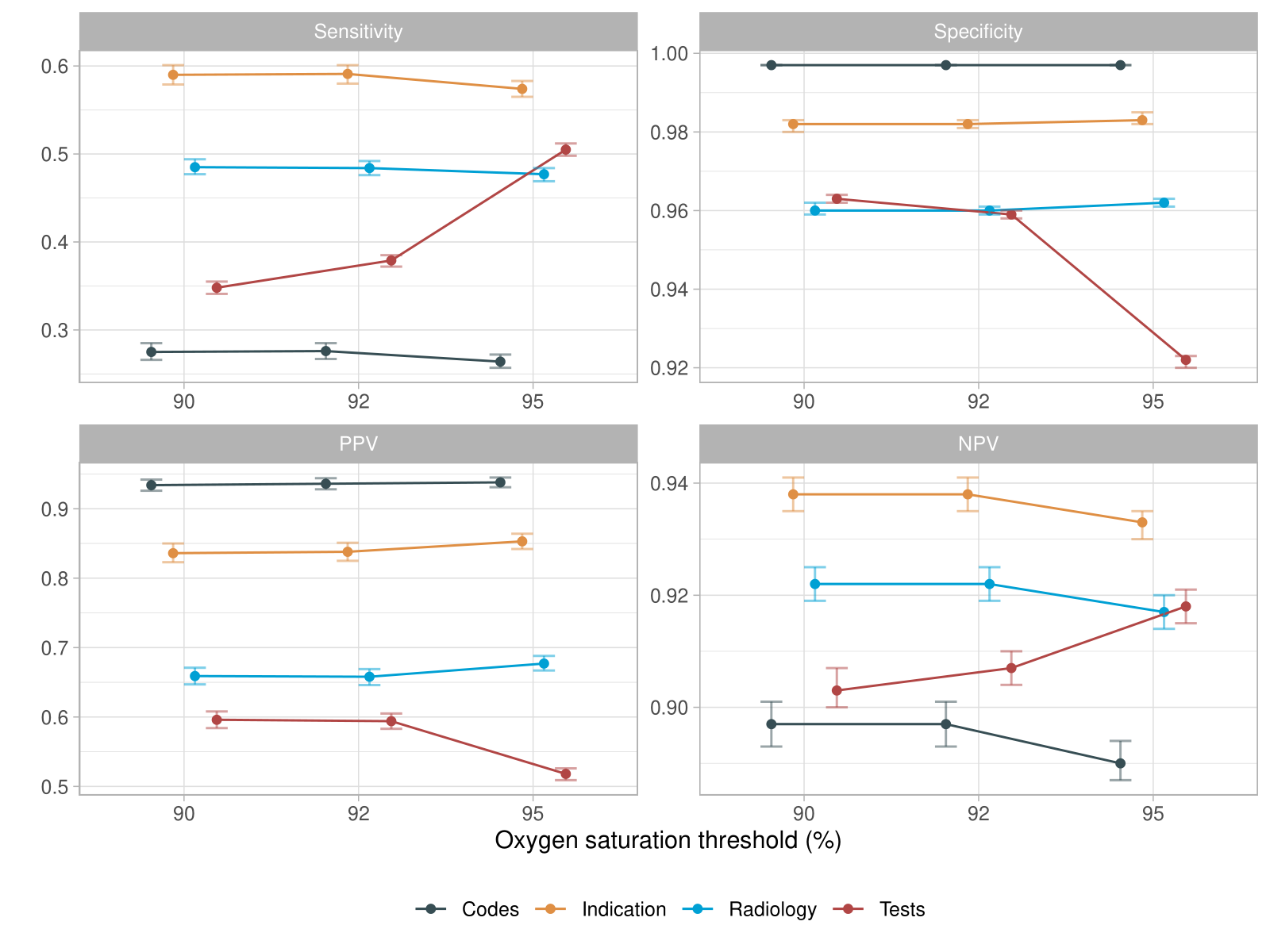

Supplement: S4 Fig — PPV: positive predictive value; NPV: negative predictive value. (TIFF) [file pdig.0000936.s004.tiff]

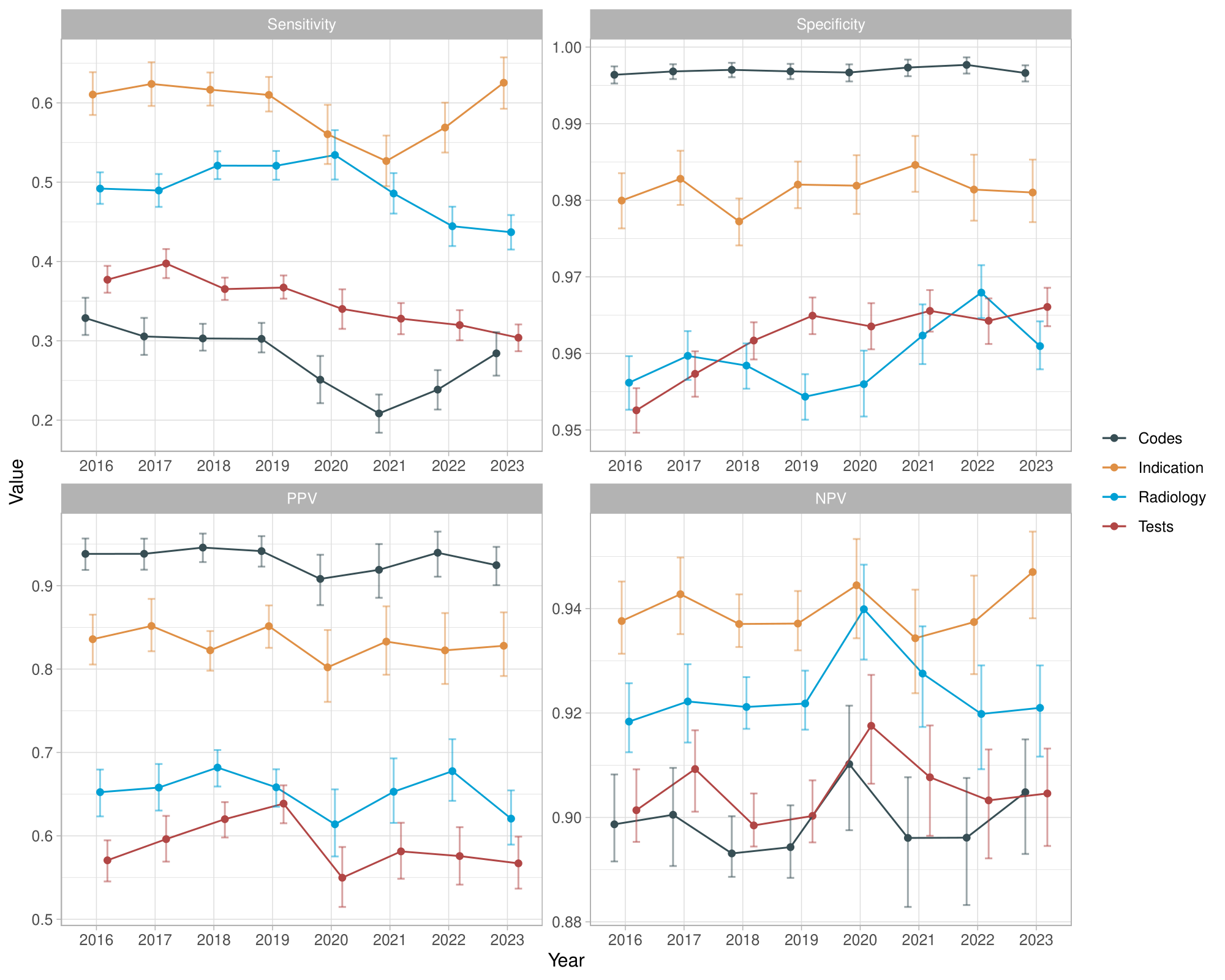

Supplement: S5 Fig — PPV: positive predictive value; NPV: negative predictive value. (TIFF) [file pdig.0000936.s005.tiff]

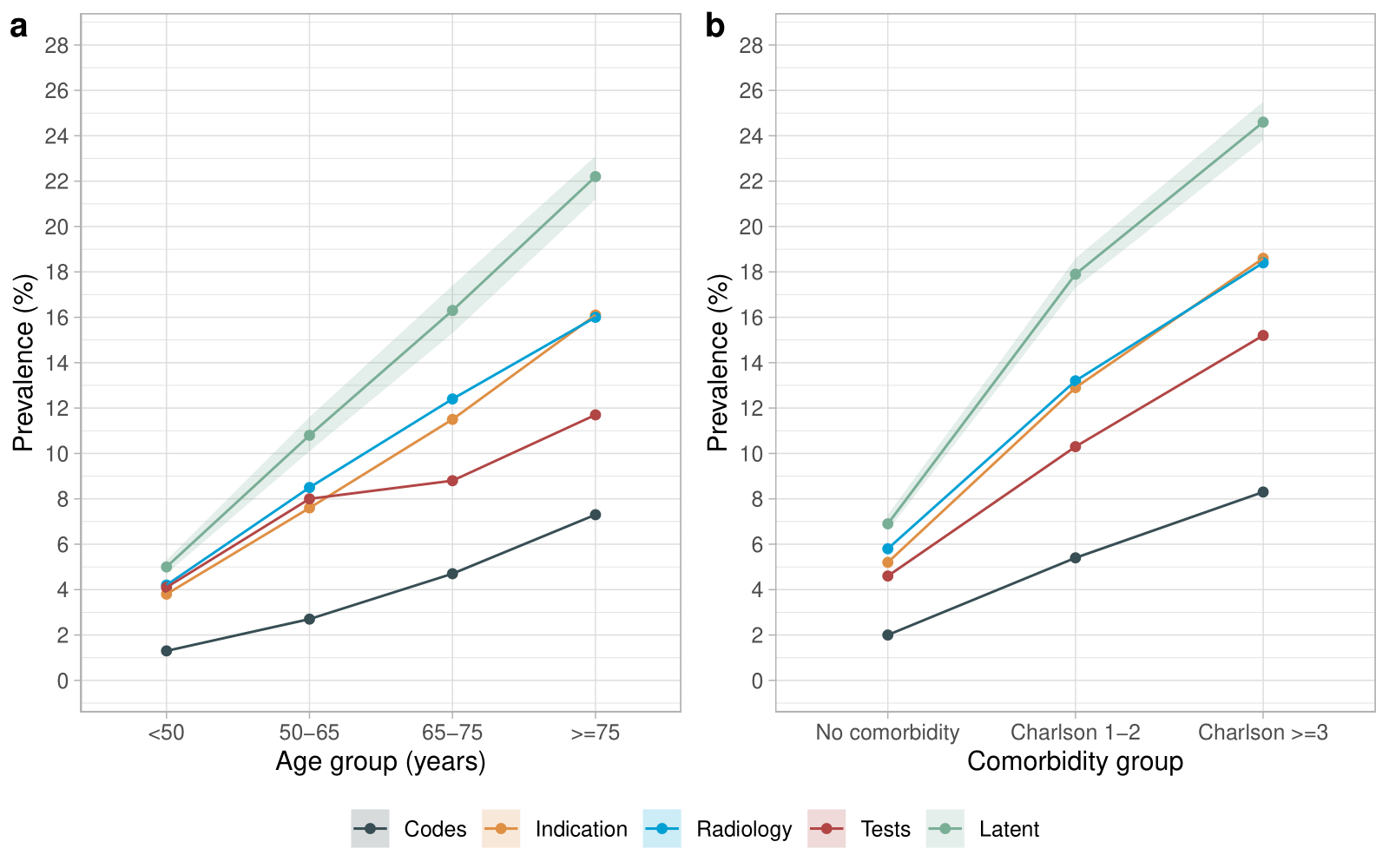

Supplement: S6 Fig — Codes: CAP primary diagnostic codes; Indication: CAP antibiotic indication; Radiology: chest X-ray report or CT scan report; Tests: shortness of breath and elevated C-reactive protein levels; Latent: latent prevalence estimated from the model. Shaded area shows the 95% credible intervals for the latent prevalence estimated from the model. (TIFF) [file pdig.0000936.s006.tiff]

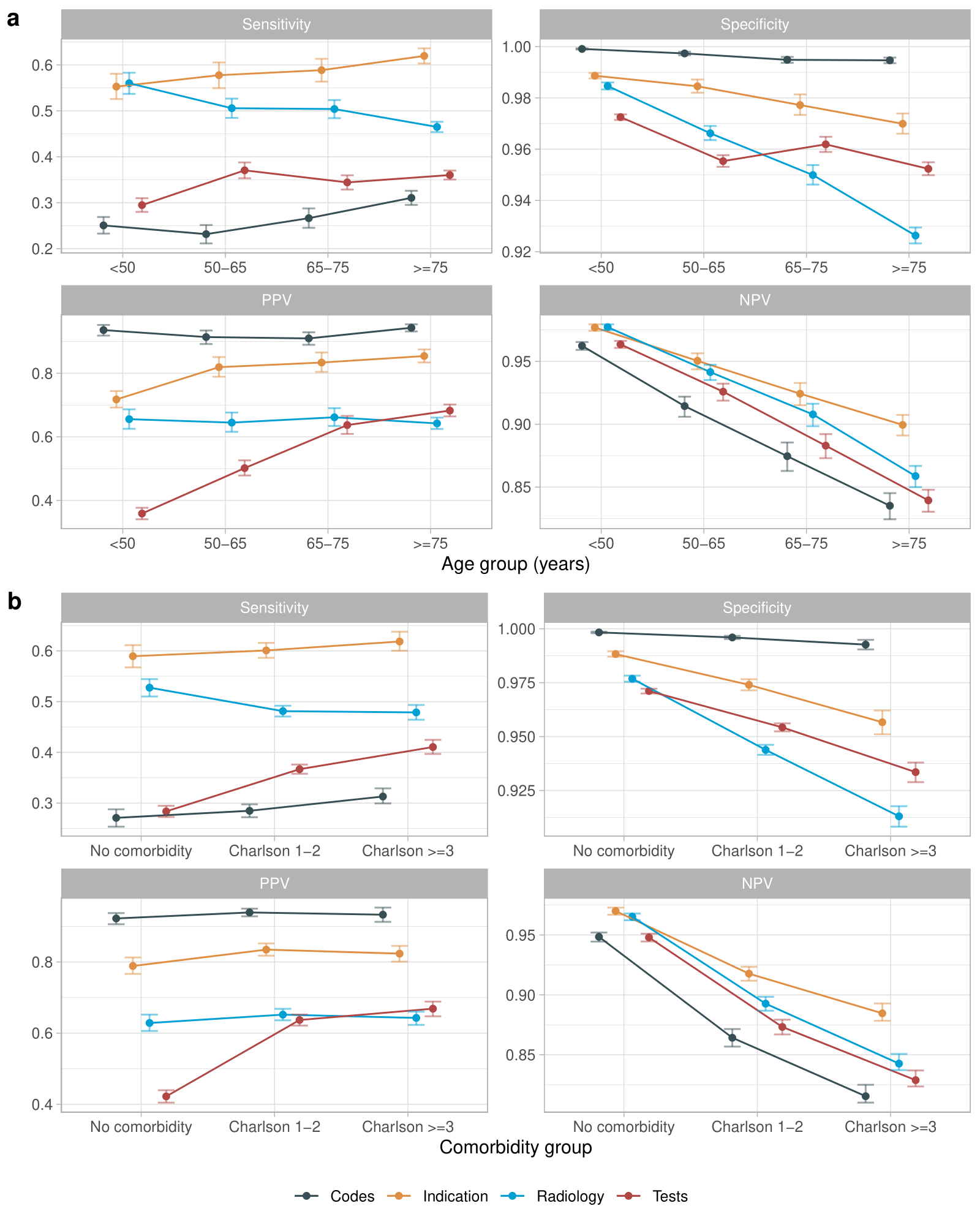

Supplement: S7 Fig — PPV: positive predictive value; NPV: negative predictive value. (TIFF) [file pdig.0000936.s007.tiff]
