# Supplementary material for: Community-acquired pneumonia identification from electronic health records in the absence of a gold standard: A Bayesian latent class analysis
Source: PLOS Digit Health. 2025 Jul 21;4(7):e0000936. doi: 10.1371/journal.pdig.0000936 (PMC12279105; doi:10.1371/journal.pdig.0000936)
Supplement: S1 Table — CAP: community-acquired pneumonia. (DOCX) [file pdig.0000936.s008.docx]

| ICD10 code | ICD10 diagnosis | Frequency | Proportion (%) |
| --- | --- | --- | --- |
| J189 | Pneumonia, unspecified organism | 9482 | 48.12 |
| J181 | Lobar pneumonia, unspecified organism | 9412 | 47.76 |
| J180 | Bronchopneumonia, unspecified organism | 280 | 1.42 |
| J159 | Bacterial pneumonia, unspecified | 144 | 0.73 |
| J13 | Pneumonia due to Streptococcus pneumoniae | 94 | 0.48 |
| J151 | Pneumonia due to Pseudomonas | 64 | 0.32 |
| J154 | Pneumonia due to other streptococci | 48 | 0.24 |
| J851 | Abscess of lung with pneumonia | 37 | 0.19 |
| J14 | Pneumonia due to Haemophilus influenzae | 36 | 0.18 |
| J152 | Pneumonia due to staphylococcus | 20 | 0.10 |
| A481 | Legionnaires disease | 19 | 0.10 |
| J150 | Pneumonia due to klebsiella pneumoniae | 15 | 0.08 |
| J188 | Other pneumonia, unspecified organism | 13 | 0.07 |
| J157 | Pneumonia due to Mycoplasma pneumoniae | 11 | 0.06 |
| J155 | Pneumonia due to Escherichia coli | 9 | 0.05 |
| J156 | Pneumonia due to other Gram-negative bacteria | 6 | 0.03 |
| J850 | Gangrene and necrosis of lung | 6 | 0.03 |
| J158 | Pneumonia due to other specified bacteria | 4 | 0.02 |
| J153 | Pneumonia due to streptococcus, group B | 3 | 0.02 |
| J168 | Pneumonia due to other specified infectious organisms | 2 | 0.01 |

**Table S1. Frequency and proportion of ICD-10 diagnostic codes for CAP in 19,705 admissions with primary diagnosis of CAP.** CAP: community-acquired pneumonia.
