# Supplementary material for: Community-acquired pneumonia identification from electronic health records in the absence of a gold standard: A Bayesian latent class analysis
Source: PLOS Digit Health. 2025 Jul 21;4(7):e0000936. doi: 10.1371/journal.pdig.0000936 (PMC12279105; doi:10.1371/journal.pdig.0000936)
Supplement: S2 Table — *Positive keywords: ‘pneumonia’, ‘consolidation’, ‘infiltrate’, ‘airspace’, ‘bronchopneumonia’, ‘infection’, ‘infective’, ‘air bronchogram’, ‘density’, ‘pneumonic’, ‘abscess’, ‘aspiration’, ‘cavity’; **Negative keywords: ‘heart failure’, ‘oedema’, ‘bronchitis’, ‘tumour’, ‘cancer’, ‘asthma’, ‘fracture’. (DOCX) [file pdig.0000936.s009.docx]

| **Algorithm** | **Labelling rules** | **Polarity** | **Coverage** | **Overlaps** | **Conflicts** |
| --- | --- | --- | --- | --- | --- |
| 1 | Positive keywords* | 1 | 0.208 | 0.083 | 0.038 |
| 2 | Clear | 0 | 0.376 | 0.288 | 0.002 |
| 3 | Normal | 0 | 0.379 | 0.302 | 0.002 |
| 4.1 | Week | 1 | 0.056 | 0.056 | 0.008 |
| 4.2 | Weeks | 1 | 0.054 | 0.054 | 0.008 |
| 5 | Negative keywords** | 0 | 0.132 | 0.093 | 0.039 |

**Table S2. Coverage, overlaps, and conflicts of the labelling rules used for identifying community-acquired pneumonia from free text chest X-ray or CT reports.** *Positive keywords: 'pneumonia', 'consolidation', 'infiltrate', 'airspace', 'bronchopneumonia', 'infection', 'infective', 'air bronchogram', 'density', 'pneumonic', 'abscess', 'aspiration', 'cavity'; **Negative keywords: 'heart failure', 'oedema', 'bronchitis', 'tumour', 'cancer', 'asthma', 'fracture'.
