# Supplementary material for: Community-acquired pneumonia identification from electronic health records in the absence of a gold standard: A Bayesian latent class analysis
Source: PLOS Digit Health. 2025 Jul 21;4(7):e0000936. doi: 10.1371/journal.pdig.0000936 (PMC12279105; doi:10.1371/journal.pdig.0000936)
Supplement: S3 Table — (DOCX) [file pdig.0000936.s010.docx]

| **Algorithm** | **Parameter** | **Primary prior set** | **Alternative prior set 1** | **Alternative prior set 2** |
| --- | --- | --- | --- | --- |
|  | CAP prevalence | Uniform (0.02, 0.15) | Uniform (0.02, 0.15) | Uniform (0.02, 0.15) |
| CAP primary diagnostic codes | Sensitivity | Mode=0.60, 95% sure >0.35; Beta(7.01, 5.01) | Mode=0.50, 95% sure >0.20; Beta(3.26, 3.26) | Uniform (0.2, 0.8) |
|  | Specificity | Mode=0.90, 95% sure >0.70; Beta(15.03, 2.56) | Mode=0.90, 95% sure >0.50; Beta(5.38, 1.49) | Uniform (0.6,1) |
| Antibiotics Indication | Sensitivity | Mode=0.80, 95% sure >0.50; Beta(7.55, 2.64) | Mode=0.70, 95% sure >0.20; Beta(2.26, 1.54) | Uniform (0.2, 0.8) |
|  | Specificity | Mode=0.85, 95% sure >0.50; Beta(6.25, 1.93) | Mode=0.90, 95% sure >0.50; Beta(5.38, 1.49) | Uniform (0.6,1) |
| Radiology report | Sensitivity | Mode=0.60, 95% sure >0.35; Beta(7.01, 5.01) | Mode=0.50, 95% sure >0.20; Beta(3.26, 3.26) | Uniform (0.2, 0.8) |
|  | Specificity | Mode=0.90, 95% sure >0.60; Beta(8.30, 1.81) | Mode=0.90, 95% sure >0.50; Beta(5.38, 1.49) | Uniform (0.6,1) |
| Test results | Sensitivity | Mode=0.60, 95% sure >0.35; Beta(7.01, 5.01) | Mode=0.50, 95% sure >0.20; Beta(3.26, 3.26) | Uniform (0.2, 0.8) |
|  | Specificity | Mode=0.90, 95% sure >0.70; Beta(15.03, 2.56) | Mode=0.90, 95% sure >0.50; Beta(5.38, 1.49) | Uniform (0.6,1) |

**Table S3. Priors for the prevalence of community-acquired pneumonia, sensitivities and specificities of the four algorithms in the Bayesian latent class model.**
