# Supplementary material for: Community-acquired pneumonia identification from electronic health records in the absence of a gold standard: A Bayesian latent class analysis
Source: PLOS Digit Health. 2025 Jul 21;4(7):e0000936. doi: 10.1371/journal.pdig.0000936 (PMC12279105; doi:10.1371/journal.pdig.0000936)
Supplement: S4 Table — (DOCX) [file pdig.0000936.s011.docx]

| **Algorithms** | | | | | **Observed frequency** | | **Predicted frequency** | | | |
| --- | --- | --- | --- | --- | --- | --- | --- | --- | --- | --- |
| **Primary codes** | **Antibiotic indication** | **Radiology report** | **Test results** |  | | **Model1** | | **Model2** | **Model3** | **Model4** |
| 1 | 1 | 1 | 0 | 5406 | | 4691 | | 5322 | 5267 | 5232 |
| 1 | 1 | 1 | 1 | 3278 | | 2868 | | 2999 | 3073 | 3094 |
| 1 | 1 | 0 | 1 | 2233 | | 2570 | | 2508 | 2598 | 2570 |
| 1 | 0 | 1 | 1 | 770 | | 1045 | | 798 | 879 | 895 |
| 0 | 1 | 1 | 1 | 3456 | | 4525 | | 3904 | 3624 | 3634 |
| 1 | 1 | 0 | 0 | 4408 | | 4208 | | 4469 | 4366 | 4404 |
| 1 | 0 | 1 | 0 | 1268 | | 1716 | | 1451 | 1351 | 1347 |
| 0 | 1 | 1 | 0 | 7473 | | 7882 | | 7263 | 7551 | 7549 |
| 0 | 1 | 0 | 1 | 5014 | | 4487 | | 4748 | 4765 | 4760 |
| 1 | 0 | 0 | 1 | 650 | | 943 | | 503 | 516 | 511 |
| 0 | 0 | 1 | 1 | 4494 | | 2662 | | 4376 | 4388 | 4379 |
| 0 | 1 | 0 | 0 | 16033 | | 16070 | | 16084 | 16062 | 16060 |
| 0 | 0 | 1 | 0 | 23300 | | 24053 | | 23331 | 23316 | 23317 |
| 1 | 0 | 0 | 0 | 1692 | | 1667 | | 1705 | 1708 | 1705 |
| 0 | 0 | 0 | 1 | 19298 | | 20107 | | 19356 | 19353 | 19354 |
| 0 | 0 | 0 | 0 | 392908 | | 392185 | | 392858 | 392866 | 392859 |

**Table S4. Cross-classified results for the observed frequency and the predicted frequency of each algorithm combination under four models in the primary analysis.**
