# Supplementary material for: Community-acquired pneumonia identification from electronic health records in the absence of a gold standard: A Bayesian latent class analysis
Source: PLOS Digit Health. 2025 Jul 21;4(7):e0000936. doi: 10.1371/journal.pdig.0000936 (PMC12279105; doi:10.1371/journal.pdig.0000936)
Supplement: S5 Table — Alternative prior information is summarised in S3 Table. (DOCX) [file pdig.0000936.s012.docx]

| **Parameter** | **Alternative prior 1** | **Alternative prior 2** |
| --- | --- | --- |
| prevalence | 0.137 (0.133-0.141) | 0.137 (0.133-0.141) |
| **Primary codes** | | |
| sensitivity | 0.275 (0.265-0.284) | 0.275 (0.265-0.284) |
| specificity | 0.997 (0.997-0.997) | 0.997 (0.997-0.997) |
| PPV | 0.934 (0.925-0.942) | 0.934 (0.925-0.942) |
| NPV | 0.897 (0.892-0.901) | 0.897 (0.892-0.901) |
| **Antibiotic indication** | | |
| sensitivity | 0.589 (0.578-0.600) | 0.589 (0.578-0.600) |
| specificity | 0.982 (0.980-0.983) | 0.982 (0.980-0.983) |
| PPV | 0.837 (0.824-0.850) | 0.837 (0.824-0.850) |
| NPV | 0.938 (0.934-0.941) | 0.938 (0.934-0.941) |
| **Radiology report** | | |
| sensitivity | 0.485 (0.477-0.493) | 0.485 (0.477-0.494) |
| specificity | 0.960 (0.959-0.962) | 0.960 (0.959-0.962) |
| PPV | 0.659 (0.647-0.672) | 0.659 (0.647-0.672) |
| NPV | 0.922 (0.918-0.925) | 0.922 (0.918-0.925) |
| **Test results** | | |
| sensitivity | 0.348 (0.341-0.354) | 0.348 (0.341-0.355) |
| specificity | 0.963 (0.962-0.964) | 0.963 (0.962-0.964) |
| PPV | 0.596 (0.585-0.608) | 0.596 (0.584-0.608) |
| NPV | 0.903 (0.900-0.907) | 0.903 (0.899-0.907) |
| **Covariance** | | |
| cov12\|D=1 | 0.066 (0.063-0.068) | 0.066 (0.063-0.068) |
| cov13\|D=1 | 0.023 (0.021-0.026) | 0.023 (0.021-0.026) |
| cov14\|D=1 | 0.009 (0.006-0.011) | 0.009 (0.006-0.011) |
| **Model comparison** | | |
| DIC | 311 | 312 |

**Table S5. Posterior predicted results under Model 3 in the primary analysis using alternative prior sets.** Alternative prior information is summarised in Table S3.
