# Supplementary material for: Community-acquired pneumonia identification from electronic health records in the absence of a gold standard: A Bayesian latent class analysis
Source: PLOS Digit Health. 2025 Jul 21;4(7):e0000936. doi: 10.1371/journal.pdig.0000936 (PMC12279105; doi:10.1371/journal.pdig.0000936)
Supplement: S6 Table — PPV: positive predictive value; NPV: negative predictive value. (DOCX) [file pdig.0000936.s013.docx]

| Parameter | Assume missing vitals and CRP are normal | Remove admissions with missing vitals and CRP | Remove admissions with missing radiology |
| --- | --- | --- | --- |
| prevalence | 0.131 (0.127-0.135) | 0.124 (0.121-0.128) | 0.217 (0.210-0.224) |
| Primary codes | |  |  |
| sensitivity | 0.285 (0.275-0.295) | 0.283 (0.273-0.293) | 0.311 (0.298-0.323) |
| specificity | 0.997 (0.996-0.997) | 0.997 (0.997-0.998) | 0.996 (0.995-0.996) |
| PPV | 0.926 (0.917-0.934) | 0.938 (0.930-0.947) | 0.952 (0.943-0.961) |
| NPV | 0.903 (0.898-0.907) | 0.907 (0.904-0.911) | 0.839 (0.831-0.847) |
| Antibiotic indication | |  |  |
| sensitivity | 0.612 (0.601-0.623) | 0.610 (0.599-0.622) | 0.634 (0.621-0.648) |
| specificity | 0.981 (0.980-0.983) | 0.981 (0.980-0.983) | 0.952 (0.949-0.955) |
| PPV | 0.831 (0.817-0.845) | 0.823 (0.809-0.836) | 0.785 (0.770-0.801) |
| NPV | 0.944 (0.941-0.947) | 0.947 (0.944-0.949) | 0.904 (0.897-0.910) |
| Radiology report |  |  |  |
| sensitivity | 0.489 (0.480-0.498) | 0.486 (0.477-0.495) | 0.573 (0.563-0.583) |
| specificity | 0.958 (0.956-0.959) | 0.957 (0.956-0.958) | 0.882 (0.879-0.885) |
| PPV | 0.635 (0.623-0.647) | 0.617 (0.605-0.628) | 0.574 (0.561-0.587) |
| NPV | 0.926 (0.923-0.929) | 0.929 (0.926-0.932) | 0.882 (0.875-0.888) |
| Test results |  |  |  |
| sensitivity | 0.246 (0.240-0.252) | 0.331 (0.323-0.338) | 0.340 (0.331-0.348) |
| specificity | 0.985 (0.985-0.986) | 0.981 (0.981-0.982) | 0.965 (0.964-0.967) |
| PPV | 0.714 (0.701-0.726) | 0.715 (0.703-0.728) | 0.731 (0.715-0.746) |
| NPV | 0.897 (0.893-0.900) | 0.912 (0.908-0.915) | 0.841 (0.834-0.848) |

**Table S6. Posterior predicted prevalence, sensitivity, specificity, PPV, and NPV under Model-3 in the sensitivity analyses dealing with missing values.** PPV: positive predictive value; NPV: negative predictive value.
