# Supplementary material for: Community-acquired pneumonia identification from electronic health records in the absence of a gold standard: A Bayesian latent class analysis
Source: PLOS Digit Health. 2025 Jul 21;4(7):e0000936. doi: 10.1371/journal.pdig.0000936 (PMC12279105; doi:10.1371/journal.pdig.0000936)
Supplement: S7 Table — PPV: positive predictive value; NPV: negative predictive value. (DOCX) [file pdig.0000936.s014.docx]

| Parameter | Oxygen saturation <90% | Oxygen saturation <92% | Oxygen saturation <95% |
| --- | --- | --- | --- |
| prevalence | 0.136 (0.133-0.140) | 0.137 (0.133-0.140) | 0.143 (0.140-0.146) |
| Primary codes |  |  |  |
| sensitivity | 0.275 (0.266-0.285) | 0.276 (0.267-0.285) | 0.264 (0.257-0.272) |
| specificity | 0.997 (0.997-0.997) | 0.997 (0.997-0.997) | 0.997 (0.997-0.997) |
| PPV | 0.934 (0.926-0.942) | 0.936 (0.928-0.944) | 0.938 (0.931-0.945) |
| NPV | 0.897 (0.893-0.901) | 0.897 (0.893-0.901) | 0.890 (0.887-0.894) |
| Antibiotic indication |  |  |  |
| sensitivity | 0.590 (0.579-0.601) | 0.591 (0.580-0.601) | 0.574 (0.565-0.583) |
| specificity | 0.982 (0.980-0.983) | 0.982 (0.981-0.983) | 0.983 (0.982-0.985) |
| PPV | 0.836 (0.823-0.850) | 0.838 (0.825-0.851) | 0.853 (0.842-0.864) |
| NPV | 0.938 (0.935-0.941) | 0.938 (0.935-0.941) | 0.933 (0.930-0.935) |
| Radiology report |  |  |  |
| sensitivity | 0.485 (0.477-0.494) | 0.484 (0.476-0.492) | 0.477 (0.469-0.484) |
| specificity | 0.960 (0.959-0.962) | 0.960 (0.959-0.961) | 0.962 (0.961-0.963) |
| PPV | 0.659 (0.647-0.671) | 0.658 (0.646-0.669) | 0.677 (0.667-0.688) |
| NPV | 0.922 (0.919-0.925) | 0.922 (0.919-0.925) | 0.917 (0.914-0.920) |
| Test results |  |  |  |
| sensitivity | 0.348 (0.341-0.355) | 0.379 (0.372-0.385) | 0.505 (0.498-0.512) |
| specificity | 0.963 (0.962-0.964) | 0.959 (0.958-0.960) | 0.922 (0.920-0.923) |
| PPV | 0.596 (0.584-0.608) | 0.594 (0.583-0.605) | 0.518 (0.509-0.526) |
| NPV | 0.903 (0.900-0.907) | 0.907 (0.904-0.910) | 0.918 (0.915-0.921) |

**Table S7. Posterior predicted prevalence, sensitivity, specificity, PPV, and NPV under Model-3 in the sensitivity analyses using different oxygen saturation thresholds.** PPV: positive predictive value; NPV: negative predictive value.
