# Supplementary material for: Community-acquired pneumonia identification from electronic health records in the absence of a gold standard: A Bayesian latent class analysis
Source: PLOS Digit Health. 2025 Jul 21;4(7):e0000936. doi: 10.1371/journal.pdig.0000936 (PMC12279105; doi:10.1371/journal.pdig.0000936)
Supplement: S8 Table — PPV: positive predictive value; NPV: negative predictive value. (DOCX) [file pdig.0000936.s015.docx]

| Parameter | 2016 | 2017 | 2018 | 2019 | 2020 | 2021 | 2022 | 2023 |
| --- | --- | --- | --- | --- | --- | --- | --- | --- |
| prevalence | 0.143 (0.134-0.150) | 0.137 (0.128-0.146) | 0.146 (0.140-0.150) | 0.144 (0.137-0.150) | 0.116 (0.105-0.128) | 0.127 (0.116-0.140) | 0.132 (0.121-0.144) | 0.128 (0.118-0.139) |
| Primary codes | |  |  |  |  |  |  |  |
| sensitivity | 0.329 (0.307-0.354) | 0.305 (0.282-0.329) | 0.303 (0.288-0.321) | 0.302 (0.285-0.323) | 0.251 (0.221-0.281) | 0.208 (0.184-0.232) | 0.238 (0.213-0.263) | 0.284 (0.256-0.311) |
| specificity | 0.996 (0.995-0.997) | 0.997 (0.996-0.998) | 0.997 (0.996-0.998) | 0.997 (0.996-0.998) | 0.997 (0.996-0.998) | 0.997 (0.996-0.998) | 0.998 (0.997-0.999) | 0.997 (0.996-0.998) |
| PPV | 0.938 (0.919-0.957) | 0.938 (0.919-0.956) | 0.946 (0.928-0.963) | 0.942 (0.923-0.959) | 0.908 (0.877-0.937) | 0.919 (0.886-0.950) | 0.940 (0.911-0.965) | 0.925 (0.901-0.947) |
| NPV | 0.899 (0.892-0.908) | 0.901 (0.891-0.909) | 0.893 (0.889-0.900) | 0.894 (0.888-0.902) | 0.910 (0.898-0.921) | 0.896 (0.883-0.908) | 0.896 (0.883-0.908) | 0.905 (0.893-0.915) |
| Antibiotic indication | |  |  |  |  |  |  |  |
| sensitivity | 0.610 (0.585-0.639) | 0.624 (0.596-0.651) | 0.617 (0.597-0.638) | 0.610 (0.589-0.633) | 0.560 (0.523-0.598) | 0.527 (0.495-0.559) | 0.569 (0.537-0.600) | 0.625 (0.593-0.657) |
| specificity | 0.980 (0.976-0.984) | 0.983 (0.979-0.986) | 0.977 (0.974-0.980) | 0.982 (0.979-0.985) | 0.982 (0.978-0.986) | 0.985 (0.981-0.988) | 0.981 (0.977-0.986) | 0.981 (0.977-0.985) |
| PPV | 0.836 (0.805-0.865) | 0.852 (0.821-0.884) | 0.823 (0.798-0.846) | 0.851 (0.825-0.876) | 0.802 (0.761-0.847) | 0.833 (0.793-0.875) | 0.822 (0.782-0.867) | 0.828 (0.792-0.868) |
| NPV | 0.938 (0.931-0.945) | 0.943 (0.935-0.950) | 0.937 (0.933-0.943) | 0.937 (0.932-0.943) | 0.944 (0.934-0.953) | 0.934 (0.924-0.944) | 0.937 (0.927-0.946) | 0.947 (0.938-0.955) |
| Radiology report | |  |  |  |  |  |  |  |
| sensitivity | 0.492 (0.473-0.512) | 0.490 (0.469-0.510) | 0.521 (0.504-0.539) | 0.521 (0.503-0.539) | 0.534 (0.503-0.566) | 0.486 (0.460-0.511) | 0.444 (0.419-0.469) | 0.437 (0.415-0.459) |
| specificity | 0.956 (0.953-0.960) | 0.960 (0.957-0.963) | 0.958 (0.955-0.961) | 0.954 (0.951-0.957) | 0.956 (0.952-0.960) | 0.962 (0.959-0.966) | 0.968 (0.965-0.972) | 0.961 (0.958-0.964) |
| PPV | 0.652 (0.623-0.680) | 0.658 (0.630-0.686) | 0.682 (0.659-0.703) | 0.658 (0.635-0.680) | 0.614 (0.575-0.656) | 0.653 (0.616-0.693) | 0.678 (0.642-0.716) | 0.621 (0.589-0.655) |
| NPV | 0.918 (0.912-0.926) | 0.922 (0.914-0.929) | 0.921 (0.917-0.927) | 0.922 (0.917-0.928) | 0.940 (0.930-0.948) | 0.928 (0.917-0.937) | 0.920 (0.909-0.929) | 0.921 (0.912-0.929) |
| Test results | |  |  |  |  |  |  |  |
| sensitivity | 0.377 (0.361-0.394) | 0.397 (0.379-0.416) | 0.365 (0.352-0.380) | 0.367 (0.353-0.382) | 0.340 (0.315-0.365) | 0.328 (0.308-0.348) | 0.320 (0.301-0.339) | 0.304 (0.287-0.321) |
| specificity | 0.953 (0.950-0.955) | 0.957 (0.954-0.960) | 0.962 (0.959-0.964) | 0.965 (0.963-0.967) | 0.964 (0.961-0.967) | 0.966 (0.963-0.968) | 0.964 (0.961-0.967) | 0.966 (0.964-0.969) |
| PPV | 0.571 (0.545-0.595) | 0.596 (0.569-0.624) | 0.620 (0.598-0.640) | 0.639 (0.615-0.661) | 0.550 (0.515-0.587) | 0.581 (0.548-0.616) | 0.576 (0.541-0.610) | 0.567 (0.537-0.599) |
| NPV | 0.901 (0.895-0.909) | 0.909 (0.901-0.917) | 0.898 (0.894-0.905) | 0.900 (0.895-0.907) | 0.918 (0.906-0.927) | 0.908 (0.896-0.918) | 0.903 (0.892-0.913) | 0.905 (0.895-0.913) |

**Table S8. Posterior predicted prevalence, sensitivity, specificity, PPV, and NPV under Model-3 in the subgroup analyses by year.** PPV: positive predictive value; NPV: negative predictive value.
