# Supplementary material for: Community-acquired pneumonia identification from electronic health records in the absence of a gold standard: A Bayesian latent class analysis
Source: PLOS Digit Health. 2025 Jul 21;4(7):e0000936. doi: 10.1371/journal.pdig.0000936 (PMC12279105; doi:10.1371/journal.pdig.0000936)
Supplement: S9 Table — PPV: positive predictive value; NPV: negative predictive value. (DOCX) [file pdig.0000936.s016.docx]

|  |  |  |  |  |  |  |  |  |  |
| --- | --- | --- | --- | --- | --- | --- | --- | --- | --- |

|  | Age group (years) | | | | Comorbidity group (Charlson score) | | |
| --- | --- | --- | --- | --- | --- | --- | --- |
| Parameter | <50 | 50-65 | 65-75 | ≥75 | 0 | 1-2 | ≥3 |
| prevalence | 0.050 (0.047-0.053) | 0.108 (0.101-0.116) | 0.163 (0.153-0.174) | 0.222 (0.212-0.231) | 0.069 (0.066-0.073) | 0.179 (0.173-0.186) | 0.246 (0.238-0.250) |
| Primary codes |  |  |  |  |  |  |  |
| sensitivity | 0.251 (0.233-0.269) | 0.232 (0.211-0.251) | 0.266 (0.245-0.288) | 0.311 (0.295-0.326) | 0.271 (0.253-0.288) | 0.285 (0.272-0.298) | 0.313 (0.299-0.329) |
| specificity | 0.999 (0.999-0.999) | 0.997 (0.997-0.998) | 0.995 (0.994-0.996) | 0.995 (0.994-0.996) | 0.998 (0.998-0.999) | 0.996 (0.995-0.997) | 0.993 (0.990-0.995) |
| PPV | 0.936 (0.919-0.952) | 0.914 (0.892-0.934) | 0.910 (0.889-0.929) | 0.943 (0.932-0.954) | 0.922 (0.906-0.938) | 0.939 (0.928-0.950) | 0.933 (0.913-0.953) |
| NPV | 0.962 (0.959-0.965) | 0.914 (0.906-0.922) | 0.875 (0.863-0.885) | 0.835 (0.824-0.845) | 0.948 (0.944-0.952) | 0.864 (0.857-0.871) | 0.815 (0.810-0.825) |
| Antibiotic indication | |  |  |  |  |  |  |
| sensitivity | 0.553 (0.526-0.581) | 0.578 (0.549-0.605) | 0.588 (0.564-0.613) | 0.619 (0.603-0.636) | 0.589 (0.567-0.611) | 0.601 (0.586-0.616) | 0.618 (0.600-0.638) |
| specificity | 0.989 (0.988-0.990) | 0.985 (0.982-0.987) | 0.977 (0.973-0.981) | 0.970 (0.966-0.974) | 0.988 (0.987-0.990) | 0.974 (0.971-0.977) | 0.957 (0.951-0.962) |
| PPV | 0.718 (0.692-0.744) | 0.819 (0.789-0.851) | 0.834 (0.804-0.865) | 0.854 (0.834-0.875) | 0.789 (0.766-0.813) | 0.835 (0.818-0.852) | 0.823 (0.801-0.845) |
| NPV | 0.977 (0.974-0.980) | 0.950 (0.944-0.956) | 0.924 (0.915-0.933) | 0.899 (0.891-0.907) | 0.970 (0.967-0.973) | 0.918 (0.912-0.923) | 0.885 (0.878-0.893) |
| Radiology report |  |  |  |  |  |  |  |
| sensitivity | 0.560 (0.537-0.583) | 0.506 (0.485-0.527) | 0.504 (0.484-0.523) | 0.465 (0.454-0.476) | 0.527 (0.510-0.544) | 0.481 (0.471-0.492) | 0.479 (0.464-0.493) |
| specificity | 0.985 (0.983-0.986) | 0.966 (0.964-0.969) | 0.950 (0.946-0.954) | 0.926 (0.923-0.929) | 0.977 (0.975-0.978) | 0.944 (0.942-0.946) | 0.913 (0.908-0.918) |
| PPV | 0.656 (0.625-0.687) | 0.645 (0.616-0.676) | 0.662 (0.634-0.690) | 0.642 (0.625-0.661) | 0.629 (0.606-0.652) | 0.652 (0.636-0.668) | 0.643 (0.623-0.660) |
| NPV | 0.977 (0.975-0.979) | 0.941 (0.935-0.947) | 0.908 (0.898-0.916) | 0.859 (0.850-0.867) | 0.965 (0.962-0.968) | 0.893 (0.887-0.898) | 0.843 (0.837-0.851) |
| Test results |  |  |  |  |  |  |  |
| sensitivity | 0.295 (0.280-0.310) | 0.370 (0.353-0.388) | 0.344 (0.329-0.360) | 0.360 (0.350-0.370) | 0.284 (0.273-0.295) | 0.367 (0.358-0.376) | 0.410 (0.397-0.425) |
| specificity | 0.973 (0.971-0.974) | 0.955 (0.953-0.958) | 0.962 (0.959-0.965) | 0.952 (0.950-0.955) | 0.971 (0.970-0.972) | 0.954 (0.952-0.956) | 0.934 (0.929-0.938) |
| PPV | 0.359 (0.341-0.377) | 0.502 (0.479-0.526) | 0.637 (0.609-0.666) | 0.683 (0.664-0.702) | 0.422 (0.405-0.439) | 0.637 (0.621-0.652) | 0.669 (0.647-0.689) |
| NPV | 0.964 (0.961-0.966) | 0.926 (0.919-0.932) | 0.883 (0.873-0.892) | 0.839 (0.830-0.848) | 0.948 (0.945-0.951) | 0.873 (0.867-0.879) | 0.829 (0.824-0.837) |

**Table S9. Posterior predicted prevalence, sensitivity, specificity, PPV, and NPV under Model-3 in the subgroup analyses by age and comorbidity.** PPV: positive predictive value; NPV: negative predictive value.
