# Supplementary material for: Community-acquired pneumonia identification from electronic health records in the absence of a gold standard: A Bayesian latent class analysis
Source: PLOS Digit Health. 2025 Jul 21;4(7):e0000936. doi: 10.1371/journal.pdig.0000936 (PMC12279105; doi:10.1371/journal.pdig.0000936)
Supplement: S1 Text — (DOCX) [file pdig.0000936.s017.docx]

## Supplementary methods

**Free text radiology reports**

The labelling rules were: 1) A report was considered ‘positive’ if it contained any of keywords indicating a possibility of CAP ('pneumonia', 'consolidation', 'infiltrate', 'airspace', 'bronchopneumonia', 'infection', 'infective', 'air bronchogram', 'density', 'pneumonic', 'abscess', 'aspiration', 'cavity'), and the keyword was not negated (negation detection was conducted using negspaCy package in Python, based on the NegEx algorithm for identifying negated findings in medical records[1]). 2) A report was considered ‘positive’ if it contained ‘weeks’, given serial imaging is often suggested to ensure a pneumonia resolves, e.g., ‘A repeat CXR in 6 weeks is suggested’. 3) A report was considered ‘negative’ if it contained ‘clear’ or ‘normal’. 4) A report was considered negative if it contained keywords indicating diseases other than CAP ('heart failure', 'oedema', 'bronchitis', 'tumour', 'cancer', 'asthma', 'fracture') and they keyword was not negated. Given these labels may be correlated or imperfect, a sophisticated label model, “Snorkel”, was used to combine the outputs of the four rules and produce a final noise-aware probabilistic label for analysis, representing the probability of CAP or its absence[2]. The label model learns weights for the labelling functions using the label matrix as input. **S2 Table** shows coverage, overlaps, and conflicts of each label.

**Priors**

We assumed a uniform (0.02, 0.15) prior for the probability of having pneumonia in EHRs based on existing literature[3–5]. Priors for sensitivity and specificity were specified using beta distributions targeting specific modes and lower limits on performance based on existing literature. For Algorithm-1, we chose a mode of 0.6/lower limit of 0.35 for sensitivity, and a mode of 0.9/lower limit of 0.7 for specificity as the prior, based on existing studies on CAP coding accuracy[3,6–9]. For Algorithm-2, since information on the sensitivity/specificity of antibiotic indication was limited, we assumed a higher sensitivity and a lower specificity than diagnostic codes with wider bounds (**S3 Table**). For Algorithm-3, existing literature on diagnosing CAP through radiology reports had relatively high sensitivity and specificity around 0.9[10,11]. However, these estimates were not directly applicable because of the different methods. We therefore assumed our algorithm had a good specificity but lower sensitivity, using a mode of 0.6 for sensitivity and 0.9 for specificity, and decreasing the lower bounds for both. For Algorithm-4, we used the same prior as Algorithm-1. We additionally selected two sets of less informative priors either with wider bounds in beta distributions or using uniform distributions for sensitivity analyses to examine the robustness of the posterior estimates (**S3 Table**).

**Computation**

The BLCM was estimated in Just Another Gibbs Sampler (JAGS) version 4.3.0[12] through the R interface “jagsUI”. JAGS is an implementation of a Markov Chain Monte Carlo (MCMC) algorithm called Gibbs sampling to sample the posterior distribution of a Bayesian model.

Three chains of 10,000 iterations were run. The first 1,000 iterations were discarded as the burn-in period to get to the stationary state. The chains were then thinned by taking every tenth sample to reduce autocorrelation. Convergence was checked by verifying the Gelman-Rubin statistic (≤1.1) and visual inspection of the MCMC trace plots, created using the ‘MCMCvis’ library[13].

95% credible intervals (Crl) were calculated from the highest posterior density intervals, and means of the posterior distributions as point estimates.

## References

1. Chapman WW, Bridewell W, Hanbury P, Cooper GF, Buchanan BG. A simple algorithm for identifying negated findings and diseases in discharge summaries. J Biomed Inform. 2001;34: 301–310. doi:10.1006/jbin.2001.1029

2. Ratner A, Bach SH, Ehrenberg H, Fries J, Wu S, Ré C. Snorkel: Rapid training data creation with weak supervision. Proceedings of the VLDB Endowment. Association for Computing Machinery; 2017. pp. 269–282. doi:10.14778/3157794.3157797

3. Aronsky D, Haug PJ, Lagor C, Dean NC. Accuracy of administrative data for identifying patients with pneumonia. American Journal of Medical Quality. 2005;20: 319–328. doi:10.1177/1062860605280358

4. Azmi S, Aljunid SM, Maimaiti N, Ali AA, Muhammad Nur A, De Rosas-Valera M, et al. Assessing the burden of pneumonia using administrative data from Malaysia, Indonesia, and the Philippines. International Journal of Infectious Diseases. 2016;49: 87–93. doi:10.1016/j.ijid.2016.05.021

5. Quan TP, Fawcett NJ, Wrightson JM, Finney J, Wyllie D, Jeffery K, et al. Increasing burden of community-acquired pneumonia leading to hospitalisation, 1998-2014. Thorax. 2016;71: 535–542. doi:10.1136/thoraxjnl-2015-207688

6. van de Garde EMW, Oosterheert JJ, Bonten M, Kaplan RC, Leufkens HGM. International classification of diseases codes showed modest sensitivity for detecting community-acquired pneumonia. J Clin Epidemiol. 2007;60: 834–838. doi:10.1016/j.jclinepi.2006.10.018

7. Williams DJ, Shah SS, Myers A, Hall M, Auger K, Queen MA, et al. Identifying pediatric community-acquired pneumonia hospitalizations: Accuracy of administrative billing codes. JAMA Pediatr. 2013;167: 851–858. doi:10.1001/jamapediatrics.2013.186

8. DeLisle S, Kim B, Deepak J, Siddiqui T, Gundlapalli A, Samore M, et al. Using the Electronic Medical Record to Identify Community-Acquired Pneumonia: Toward a Replicable Automated Strategy. PLoS One. 2013;8. doi:10.1371/journal.pone.0070944

9. Sukanya C. Validity of Principal Diagnoses in Discharge Summaries and ICD-10 Coding Assessments Based on National Health Data of Thailand. Healthc Inform Res. 2017;23: 293. doi:10.4258/hir.2017.23.4.293

10. Dublin S, Baldwin E, Walker RL, Christensen LM, Haug PJ, Jackson ML, et al. Natural language processing to identify pneumonia from radiology reports. Pharmacoepidemiol Drug Saf. 2013;22: 834–841. doi:10.1002/pds.3418

11. Liu V, Clark MP, Mendoza M, Saket R, Gardner MN, Turk BJ, et al. Automated identification of pneumonia in chest radiograph reports in critically ill patients. BMC Med Inform Decis Mak. 2013;13. doi:10.1186/1472-6947-13-90

12. Plummer M. JAGS Version 3.4.0 user manual. 2013.

13. Youngflesh C. MCMCvis: Tools to Visualize, Manipulate, and Summarize MCMC Output. J Open Source Softw. 2018;3: 640. doi:10.21105/joss.00640
