# Supplementary material for: Community-acquired pneumonia identification from electronic health records in the absence of a gold standard: A Bayesian latent class analysis
Source: PLOS Digit Health. 2025 Jul 21;4(7):e0000936. doi: 10.1371/journal.pdig.0000936 (PMC12279105; doi:10.1371/journal.pdig.0000936)
Supplement: S2 Text — (DOCX) [file pdig.0000936.s018.docx]

## Supplementary results

In sensitivity analyses assuming missing vital signs and CRP measurements indicating absence of CAP, 22,133 (4.5%) admissions had shortness of breath and elevated CRP levels at admission. The estimated CAP prevalence was 13.1% (95% Crl 12.7-13.5%), with similar sensitivity and specificity for diagnostic codes, antibiotic indication, and radiology to the primary analysis (**S6 Table**). The sensitivity of shortness of breath and elevated CRP was lower than the primary analysis (0.246, 0.240-0.252), but specificity was higher (0.985, 0.985-0.986). In sensitivity analyses excluding admissions with missing vital signs and CRP measurements, the estimated prevalence was 12.4% (95% Crl 12.1-12.8%), with similar sensitivity, specificity, PPV, and NPV to the primary analysis (**S6 Table**). In sensitivity analyses excluding admissions with missing radiology scans, the estimated prevalence increased to 21.7% (95% Crl 21.0-22.4%), with slightly higher sensitivity and lower specificity for diagnostic codes, antibiotic indication, and radiology approaches compared to primary analyses (**S6 Table**).
